# LncRNA-FKBP1C regulates muscle fiber type switching by affecting the stability of MYH1B

**DOI:** 10.1038/s41420-021-00463-7

**Published:** 2021-04-09

**Authors:** Jia-ao Yu, Zhijun Wang, Xin Yang, Manting Ma, Zhenhui Li, Qinghua Nie

**Affiliations:** 1 State Key Laboratory for Conservation and Utilization of Subtropical Agro-bioresources & Lingnan Guangdong Laboratory of Agriculture, College of Animal Science, South China Agricultural University, Guangzhou, Guangdong China; 2 Guangdong Provincial Key Lab of Agro-Animal Genomics and Molecular Breeding, and Key Laboratory of Chicken Genetics, Breeding and Reproduction, Ministry of Agriculture, Guangzhou, China; 3 National-Local Joint Engineering Research Center for Livestock Breeding, Guangzhou, China

**Keywords:** Epigenetics, Cell growth

## Abstract

Long non-coding RNAs (lncRNAs) are well-known to participate in a variety of important regulatory processes in myogenesis. In our previous RNA-seq study (accession number GSE58755), we found that lncRNA-FKBP1C was differentially expressed between White Recessive Rock (WRR) and Xinghua (XH) chicken. Here, we have further demonstrated that lncRNA-FKBP1C interacted directly with MYH1B by biotinylated RNA pull-down assay and RNA immunoprecipitation (RIP). Protein stability and degradation experiments identified that lncRNA-FKBP1C enhanced the protein stability of MYH1B. Overexpression of lncRNA-FKBP1C inhibited myoblasts proliferation, promoted myoblasts differentiation, and participated in the formation of skeletal muscle fibers. LncRNA-FKBP1C could downregulate the fast muscle genes and upregulate slow muscle genes. Conversely, its interference promoted cell proliferation, repressed cell differentiation, and drove the transformation of slow-twitch muscle fibers to fast-twitch muscle fibers. Similar results were observed after knockdown of the *MYH1B* gene, but the difference was that the *MYH1B* gene had no effects on fast muscle fibers. In short, these data demonstrate that lncRNA-FKBP1C could bound with MYH1B and enhance its protein stability, thus affecting proliferation, differentiation of myoblasts and conversion of skeletal muscle fiber types.

## Introduction

Skeletal muscle is one of the most dynamic tissues involved in a variety of biological processes^1^, and the growth and development of skeletal muscle are essential for maintaining skeletal muscle function^2^. Skeletal muscle dysfunction can lead to a variety of human muscle diseases, including muscle dystrophy, type 2 diabetes, cardiomyopathic disease, and other metabolic disorders^3–7^. Skeletal muscle is composed of various muscle fibers types [including type I and type II (IIa, IIb, and IIx) muscle fibers] that are different in function, biochemical characteristics, and morphological characteristics^8–10^. Type I fibers have higher mitochondrial and lower glycogen and glucose contents, while type II fibers have a higher glycolytic capacity. Previous research has reported that the ratio of muscle fiber types could affect meat quality^11–13^. In addition, skeletal muscle is dynamic muscle tissue, the fast muscle fiber subtype (type II) and the slow muscle fiber subtype (type I) can mutually transform regulated by noncoding RNAs^2,14,15^.

In the past, lncRNAs genes had been regarded as junk DNA. However, with the continuous advancement of sequencing technology, lncRNA have been found to participate in a variety of important regulatory processes^16–19^. LncRNAs are a type of RNA molecule whose transcript length exceeds 200 nt and does not encode protein in common^20,21^. Recent studies have demonstrated that lncRNAs play an important regulatory role in the skeletal muscle growth, development^22–24^, and muscle fibers conversion of livestock. Moreover, it has been reported that lncRNA can be an important participant in DNA methylation regulation^25,26^. LncRNAs have been reported to carry out diverse functions in *trans*^19,27^. Researchers found that non-coding RNA could play a role by binding to proteins and affecting their stability^28^.

*MYH1B*, also known as *MYH3*, is mainly expressed in various developmental stages, including the embryonic stage of skeletal muscle^29^. In addition, coding mutations in the *MYH3* gene can cause muscle development disorders in humans^30,31^. *MYH3* gene can regulate not only muscle fiber type conversion but also adipogenesis in skeletal muscle^32^. However, few reports have been made about *MYH1B* in poultry skeletal muscle development.

In our previous study, we found that the lncRNA-FKBP1C was differentially expressed between breast muscle tissues of Recessive White Rock (WRR) and Xinghua Chickens (XH). This result suggested that lncRNA-FKBP1C might affect chicken muscle growth. In this study, we explored the regulation of lncRNA-FKBP1C in chicken skeletal muscle development. We concluded that lncRNA-FKBP1C could bind to MYH1B and play a role by affecting its stability. LncRNA-FKBP1C also could regulate myoblast proliferation and differentiation, and induce the slow-twitch muscle phenotype.

## Results

### cDNA sequence, protein-encoding ability, and expression level of lncRNA-FKBP1C

**Fig. 1:**
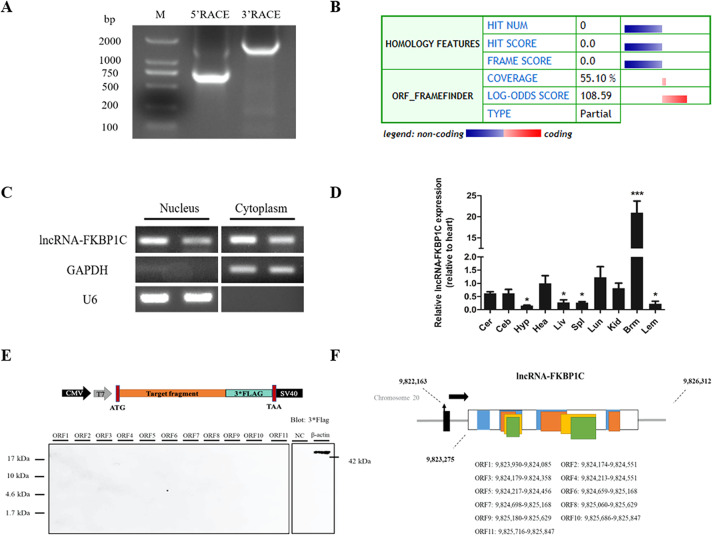
Identification of lncRNA-FKBP1C. **A** Results of 5′ RACE and 3′ RACE. M, DL2000 Marker; 5′ RACE product, 690 bp; 3′ RACE product, 1465 bp. **B** Analysis obtained from the Coding Potential Calculator (http://cpc.cbi.pku.edu.cn/) based on evolutionary conservation and ORF attributes. **C** LncRNA-FKBP1C is localized in the cytoplasm and nucleus of chicken primary myoblasts. GAPDH and U6 serve as cytoplasmic and nuclear localization controls, respectively. **D** XH chicken tissue expression profiles of lncRNA-FKBP1C. Cer, cerebrum; Ceb, cerebellum; Hyp, hypothalamus; Pit, pituitary; Hea, heart; Liv, liver; Spl, spleen; Lun, lung; Kid, kidney; Brm, breast muscle; Lem, leg muscle. **E** Western blot analysis of the coding ability of lncRNA-FKBP1C. The possible ORF of lncRNA-FKBP1C was cloned into the eukaryotic expression vector pcDNA3.1-3xFlag. Untransfected DF-1 cells were used as a negative control (NC) and DF-1 cells transfected with pcDNA3.1-3xFlag-β-actin were used as a positive control. The upper panel shows the model target fragment in the pcDNA3.1-3xFlag vector. The lower left panel is the blot of ORFs 1–11, and the lower right panel is the blot of the NC and β-actin. **F** The location of 11 potential ORFs in lncRNA-FKBP1C. 11 potential ORFs are arranged in order according to their positions. Results are shown as mean ± S.E.M. and the data are representative of at least three independent assays. Independent sample t-test was used to analyze the statistical differences between groups (*P < 0.05, ***P < 0.001).

We used RACE analysis to obtain the 5’ and 3’ ends of lncRNA-FKBP1C (Fig. 1A). We used the Coding Potential Calculator to predict the protein-encoding ability of lncRNA-FKBP1C and the result suggested a high coding potential (Fig. 1B). We analyzed the protein-encoding ability of eleven potential ORFs of lncRNA-FKBP1C by Western blot to verify this prediction. We found that lncRNA-FKBP1C is a lncRNA without protein-encoding potential (Fig. 1E, F). The NCBI BLAST indicated that lncRNA-FKBP1C was 3, 038 bp long, located on Chromosome 20 and spanned from 9,823,275-9,826,312, and comprised an intron. We further investigated the subcellular localization of lncRNA-FKBP1C, and the RT-PCR results confirmed that it is an RNA molecule present in the cytoplasm and nucleus (Fig. 1C). We also measured lncRNA-FKBP1C expression from different tissues of 7-week-old XH chickens using qRT-PCR, the results showed that lncRNA-FKBP1C was highly expressed in breast muscle (Fig. 1D).

### LncRNA-FKBP1C represses the proliferation of myoblasts

**Fig. 2:**
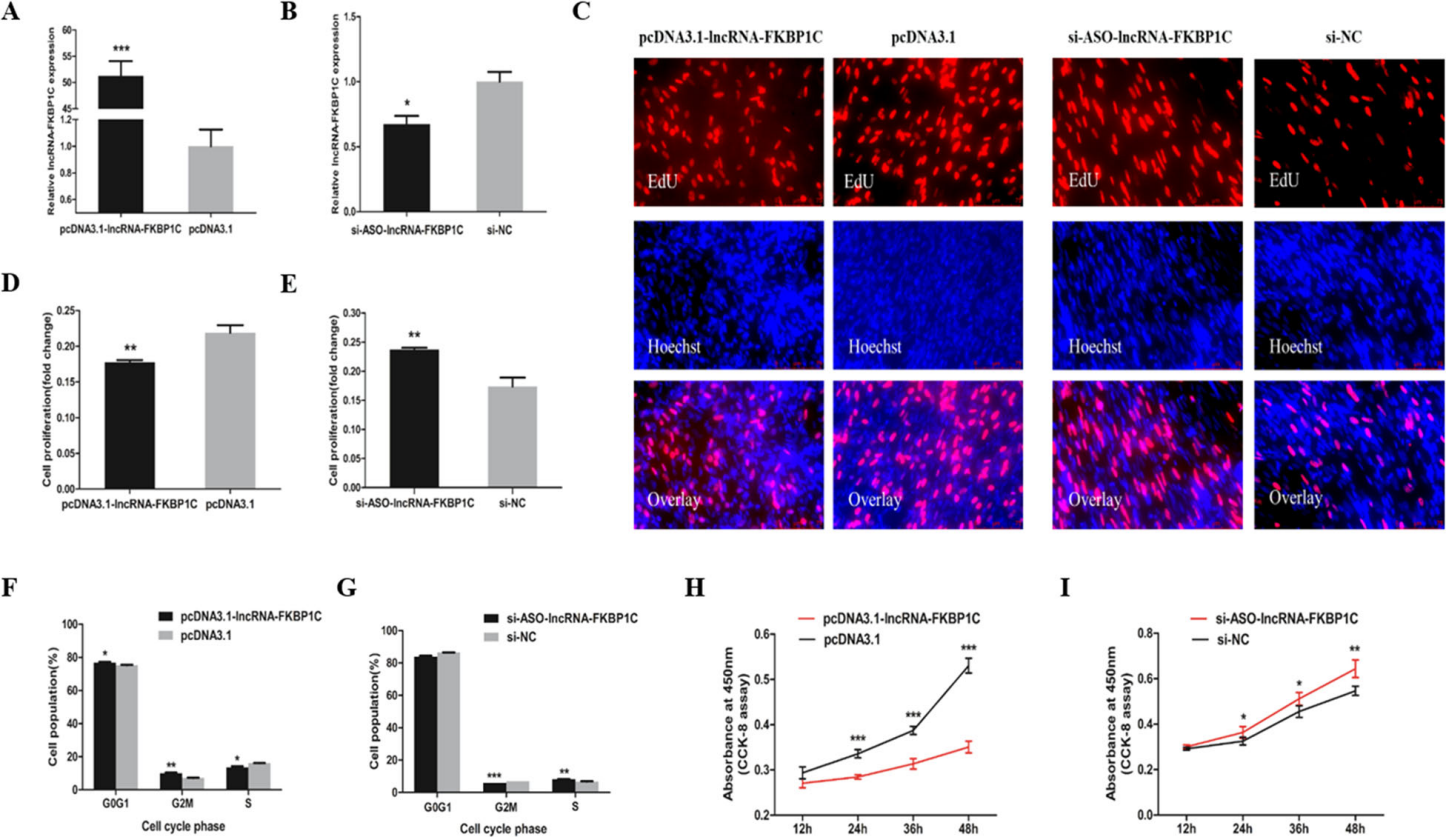
LncRNA-FKBP1C represses cell proliferation. **A, B** LncRNA-FKBP1C relative expression in CPM cells after transfection with the listed nucleic acids. **C** EdU proliferation assays for the cells after transfection. **D, E** The proliferation rate of CPM cells transfected with pcDNA3.1-lncRNA-FKBP1C or si-ASO-lncRNA-FKBP1C according to the statistical results of **C**, and the proliferation rate was calculated by the ratio of the number of EdU-stained cells to the number of Hoechst 33342-stained cells. **F, G** The results of cell cycle analysis after transfection. **H, I** The statistical results of CCK-8 assays. Results are shown as mean ± S.E.M. and the data are representative of at least three independent assays. Independent sample t-test was used to analyze the statistical differences between groups (*P < 0.05, **P < 0.01, ***P < 0.001).

To unveil the function of lncRNA-FKBP1C in myoblasts, we transfected the lncRNA-FKBP1C overexpression vector and its small interference RNA (siRNA) into the CPM cells to assess its effect on cell proliferation (Fig. 2A, B). According to the EdU assay, the overexpression of lncRNA-FKBP1C significantly hampered cell proliferation in CPM (Fig. 2C, D) and after lncRNA-FKBP1C knockdown the results were opposite (Fig. 2C, E). LncRNA-FKBP1C overexpression also significantly increased the number of G0/G1 cells, and the number of S phase cells was lower than the control group. Conversely, the cell cycle changes showed the opposite trend with lncRNA-FKBP1C interference (Fig. 2F, G). Moreover, we found that lncRNA-FKBP1C overexpression reduced cell viability by using the CCK-8 assay (Fig. 2H). After the lncRNA-FKBP1C knockdown, more cells were detected than the control group (Fig. 2I). Collectively, these data revealed that lncRNA-FKBP1C represses the proliferation of myoblasts.

### LncRNA-FKBP1C promotes the differentiation of myoblasts

**Fig. 3:**
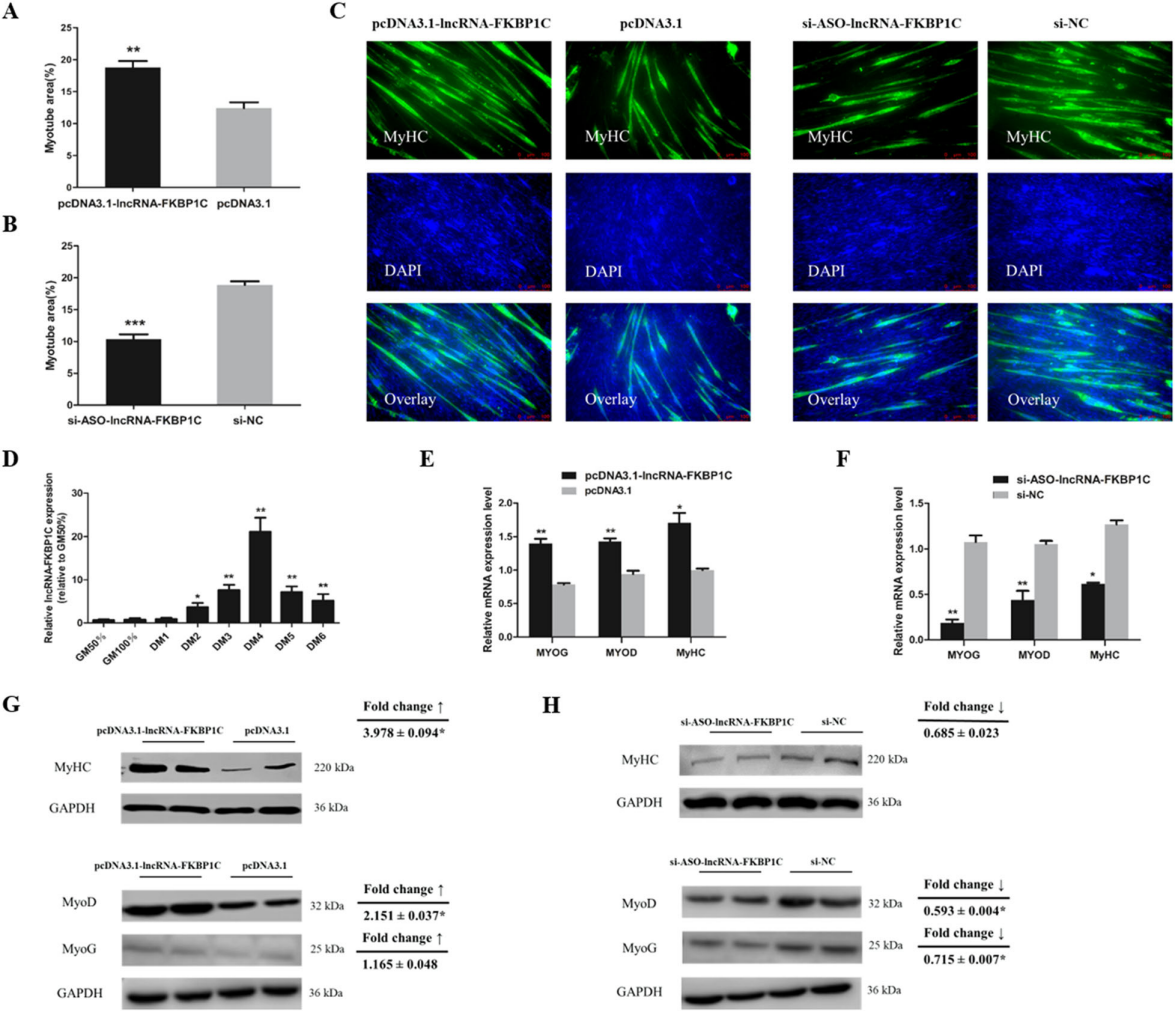
LncRNA-FKBP1C promotes cell differentiation. **A, B** Myotube area (%) of CPM cells transfected with pcDNA3.1-lncRNA-FKBP1C, pcDNA3.1, si-ASO-lncRNA-FKBP1C or si-NC. **C** MyHC Immunofluorescence staining of CPM cells. **D** The relative expression of lncRNA-FKBP1C during CPM differentiation. **E, F** The relative expression of *MyoD, MyoG*, and *MyHC*. **G, H** The protein expression of MyHC, MyoD, MyoG were determined by Western blotting in CPM cells. Results are shown as mean ± S.E.M. and the data are representative of at least three independent assays. Independent sample t-test was used to analyze the statistical differences between groups (*P < 0.05, **P < 0.01, ***P < 0.001).

The MyHC immunofluorescence staining results showed that lncRNA-FKBP1C overexpression significantly increased the area of myotubes (Fig. 3A, C), while knockdown of lncRNA-FKBP1C could prevent the formation of myotubes (Fig. 3B, C). GM stands for myoblasts in the proliferative stage, and DM1-DM6 stands for myoblasts that successfully induced differentiation from day 1 to day 6 (at different time points). The expression of lncRNA-FKBP1C was gradually increased until DM4 and then decreased (Fig. 3D) during the induction of differentiation of myoblasts. After lncRNA-FKBP1C overexpression, we found that the expression of *MyoD*, *MyoG*, and *MyHC* (myoblast differentiation marker genes) were remarkably increased. However, the knockdown of lncRNA-FKBP1C had an opposite effect on these genes (Fig. 3E, F). The protein level of MyHC, MyoD, MyoG were increased while transfected with lncRNA-FKBP1C overexpression vector by western blot analysis. However, after lncRNA-FKBP1C interference, the results were the opposite (Fig. 3G, H). In short, lncRNA-FKBP1C could promote myoblast differentiation.

### LncRNA-FKBP1C participates in the formation of skeletal muscle fibers

**Fig. 4:**
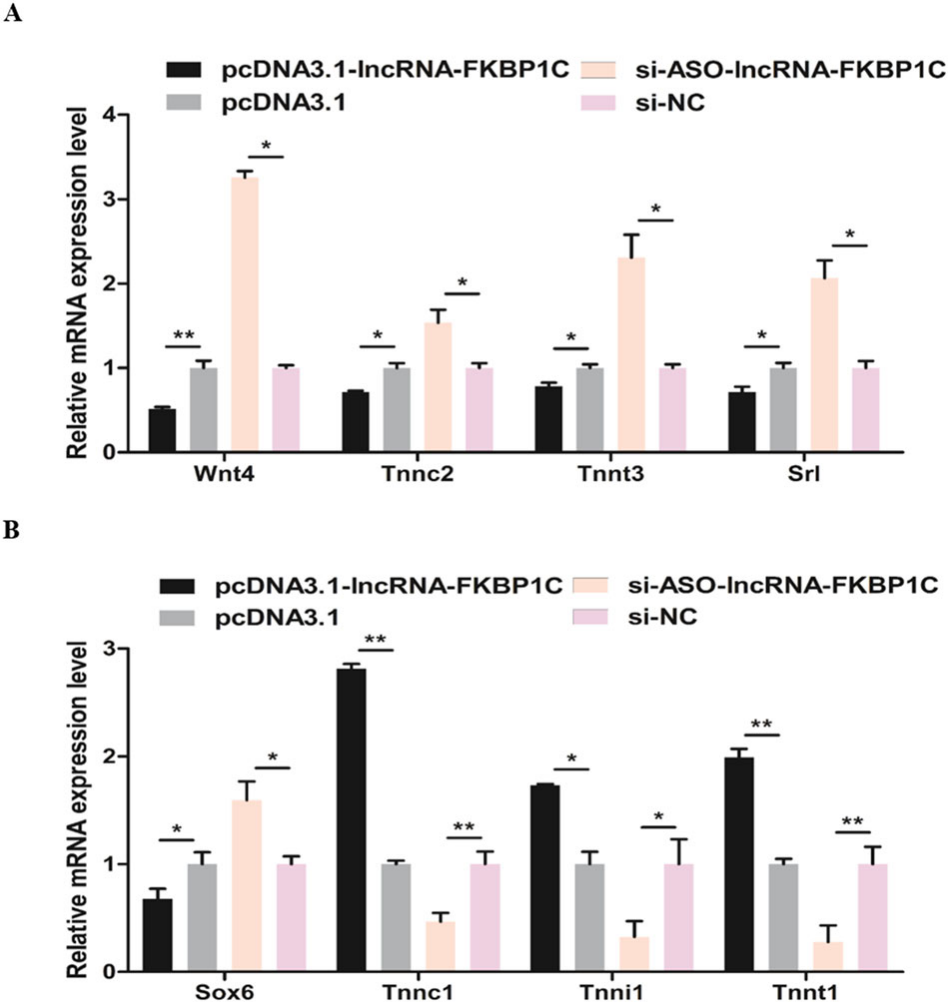
LncRNA-FKBP1C participates in the formation of skeletal muscle fibers. **A, B** The relative expression of several fast muscle genes and slow muscle genes induced by lncRNA-FKBP1C overexpression and interference in CPM cells. Results are shown as mean ± S.E.M. and the data are representative of at least three independent assays. Independent sample *t*-test was used to analyze the statistical differences between groups (*P < 0.05, **P < 0.01).

Since lncRNA-FKBP1C was differentially expressed in breast and leg muscles of 7-week-old XH chickens, we suspected that lncRNA-FKBP1C may affect the conversion of skeletal muscle fiber types. So we detected the relative mRNA level of a series of fast muscle genes (including *Wnt4*, *Tnnc2*, *Tnnt3*, and *Srl*) and a series of slow muscle genes (including *Sox6*, *Tnnc1*, *Tnni1*, and *Tnnt1*) (Fig. 4A, B). After lncRNA-FKBP1C overexpression, the expression of fast muscle genes was decreased significantly and the expression of slow muscle genes was increased. The results of lncRNA-FKBP1C interference showed that it could drove the transformation of slow-twitch muscle fibers to fast-twitch muscle fibers.

### LncRNA-FKBP1C interacts directly with MYH1B and enhances its protein stability

**Fig. 5:**
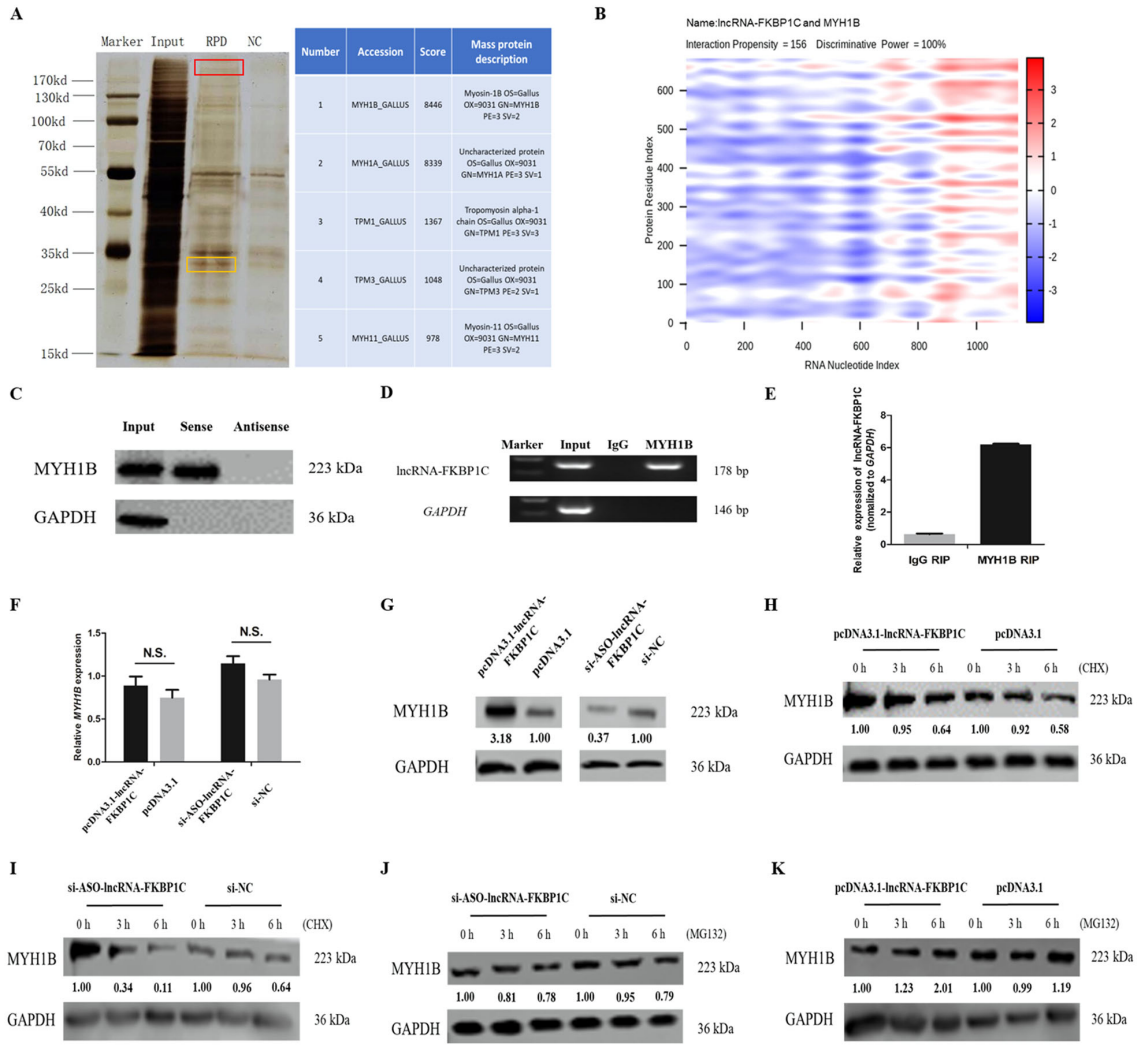
LncRNA-FKBP1C interacts directly with MYH1B and increases the protein level of MYH1B by enhancing its protein stability. **A** RNA pulldown silver stained image and binding proteins screened by mass spectrometry. Red box indicates MYH1A, MYH1B and MYH11 proteins; yellow box indicates TPM1 and TPM3 proteins. **B** Prediction of the interaction between LncRNA-FKBP1C and MYH1B by using catRAPID (http://service.tartaglialab.com/page/catrapid_group). **C, D** Interaction between LncRNA-FKBP1C and MYH1B as determined by biotin-labeled RNA pulldown and RIP. **E** The qPCR result of RIP. **F, G** The mRNA and protein expression of *MYH1B* induced by LncRNA-FKBP1C overexpression and interference in CPM cells (N.S. is the abbreviation for not significant). **H** The CPM cells after 48 h transfected with pcDNA3.1-lncRNA-FKBP1C and pcDNA3.1 incubated with the protein synthesis inhibitor CHX (1 mg/mL) for 0, 3 or 6 h. **I** The CPM cells after 48 h transfected with si-ASO-lncRNA-FKBP1C and si-NC incubated with the protein synthesis inhibitor CHX (1 mg/mL) for 0, 3 or 6 h. **J** The CPM cells after 48 h transfected with si-ASO-lncRNA-FKBP1C and si-NC incubated with MG-132 (100 μmol/L) for 0, 3 or 6 h. **K** The CPM cells after 48 h transfected with pcDNA3.1-lncRNA-FKBP1C and pcDNA3.1 incubated with MG-132 (100 μmol/L) for 0, 3 or 6 h.

To further explore the molecular mechanism of lncRNA-FKBP1C, we analyzed the localization of lncRNA-FKBP1C and found that this lncRNA was expressed in the nucleus. Also, lncRNAs have been proven to regulate the binding proteins in *trans*^19^. Thus, we hypothesized that lncRNA-FKBP1C may function by regulating its RNA-binding proteins. To certify this hypothesis, we carried out a biotinylated RNA pull-down assay to identify proteins associated with lncRNA-FKBP1C in CPM cells. RNA-binding proteins were analyzed by silver staining and mass spectrometry (Fig. 5A). Next, we selected these five most abundant proteins (including MYH1B, MYH1A, TPM1, TPM3, and MYH11) from the mass spectrometry result to verify whether they could interact with lncRNA-FKBP1C. The results showed that MYH1B was predicted to have extremely high possibilities combined with lncRNA-FKBP1C using catRAPID graphic (Fig. 5B). To further determine this specific interaction in vitro, we once again performed a biotinylated RNA pull-down assay to obtain the protein products by using biotinylated lncRNA-FKBP1C with biotinylated antisense RNA as the negative control. MYH1B was detected by Western blot assay from the obtained protein products by biotinylated RNA pull-down assays in primary myoblasts (Fig. 5C). Meanwhile, this interaction was further verified with RNA immunoprecipitation (RIP) of MYH1B. As expected, MYH1B pulled down lncRNA-FKBP1C in CPM cell lysates, revealing lncRNA-FKBP1C could interact with MYH1B in vivo (Fig. 5D, E). To sum up, lncRNA-FKBP1C interacts specifically with MYH1B in CPM cells in vitro and in vivo.

Next, we tried to explore the influence of lncRNA-FKBP1C on MYH1B. The relative mRNA expression of *MYH1B* and its protein level was detected after lncRNA-FKBP1C overexpression or knockdown. qRT-PCR results indicated that lncRNA-FKBP1C did not affect MYH1B mRNA level (Fig. 5F). However, we found a significant upregulation of MYH1B protein with overexpression of lncRNA-FKBP1C in myoblasts, while lncRNA-FKBP1C knockdown showed the opposite trend (Fig. 5G). Based on these data, we thought that lncRNA-FKBP1C may bind to MYH1B to affect its biological function at the translational or posttranslational level. To verify these hypotheses, we used protein synthesis inhibitor cycloheximide (CHX) and proteasome inhibitor MG-132 to process the CPM cells that had overexpressed or knocked-down of lncRNA-FKBP1C. CHX can decrease the expression of proteins by inhibiting protein synthesis and MG-132 mainly inhibits protein degradation by inhibiting ubiquitination-proteasome dependent degradation pathway. The western blot assays showed that overexpression of lncRNA-FKBP1C slowed down a decrease in the expression of MYH1B proteins under the treatment of CHX compared with those in control groups, which meant that overexpression of lncRNA-FKBP1C counteracted the inhibitory effect of CHX on MYH1B protein (Fig. 5H). At the same time, interference with lncRNA-FKBP1C aggravated the decline of MYH1B protein expression level (Fig. 5I). In addition, MG-132 rescued the inhibitory effect of lncRNA-FKBP1C knockdown on MYH1B protein after 6 h of treatment (Fig. 5J), and overexpression of lncRNA-FKBP1C under the treatment of MG-132 can increase the protein expression level of MYH1B (Fig. 5K). In summary, these data demonstrated that lncRNA-FKBP1C specifically interacts with MYH1B and upregulates its protein level by enhancing its protein stability.

### *MYH1B* regulates cell proliferation and differentiation of myoblast and also induces the slow-twitch muscle phenotype

**Fig. 6:**
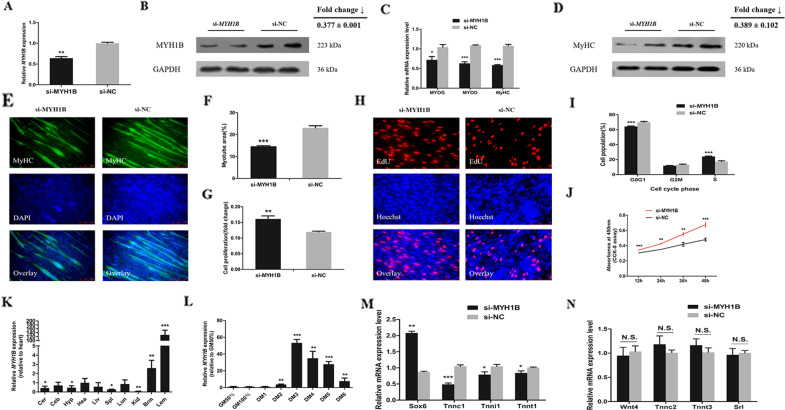
*MYH1B* represses cell proliferation, promotes cell differentiation and also induces the slow-twitch muscle phenotype. **A** The relative expression of *MYH1B* in CPM cells after transfection with si-*MYH1B* or si-NC. **B** The protein expression of MYH1B was determined by Western blotting in CPM cells. **C** The relative expression of *MyoD*, *MyoG*, and *MyHC*. **D** The protein expression of MYHC was determined by Western blotting in CPM cells. **E** MyHC Immunofluorescence staining of CPM cells after *MYH1B* interference. **F** Myotube area (%) of CPM cells. **G** The proliferation rate of CPM cells. **H** EdU proliferation assays for the cells after *MYH1B* interference. **I** The results of cell cycle analysis after transfection. **J** The statistical results of CCK-8 assays. **K** XH chicken tissue expression profiles of *MYH1B*. **L** The relative expression of *MYH1B* during CPM differentiation. **M** The relative expression of several slow muscle genes induced by MYH1B interference in CPM cells. **N** The relative expression of several fast muscle genes induced by MYH1B interference in CPM cells. In panels, results are shown as mean ± S.E.M. and the data are representative of at least three independent assays. Independent sample t-test was used to analyze the statistical differences between groups (*P < 0.05, **P < 0.01, ***P < 0.001).

To investigate the function of *MYH1B*, we detected the mRNA and protein expression of *MYH1B* after interference with *MYH1B* in primary myoblasts (Fig. 6A, B). *MYH1B* knockdown in myoblasts remarkably down-regulated the relative mRNA expression of *MyoD*, *MyoG*, and *MyHC* (Fig. 6C), and decreased the myotube area as showed in the MyHC immunofluorescence staining results (Fig. 6E, F). According to western blot analysis, the protein level of MyHC decreased after *MYH1B* knockdown (Fig. 6D). In CPM cells, the interference of *MYH1B* significantly increased the EdU stained positive cells (Fig. 6G, H). This also resulted in a significant decrease in the number of cells in G0/G1 phase, and a significant increase in the number of S-phase cells by flow cytometric analysis (Fig. 6I). Furthermore, the results of CCK-8 assay showed that *MYH1B* knockdown promoted cell proliferation (Fig. 6J). According to the tissue expression profiles and for *MYH1B*, we found it was highly expressed in breast and leg muscles of 7-week-old XH chickens (Fig. 6K). The relative mRNA expression of *MYH1B* increased abruptly at DM3 and then decreased at the stage of proliferation and differentiation in CPM (Fig. 6L). As described above, these data demonstrated that *MYH1B* inhibited cell growth and proliferation and promoted cell differentiation of myoblast.

As *MYH3* (called *MYH1B* in chickens) has been reported to influence the myofiber composition in mice, we examined the relative mRNA level of fast-type muscle fiber-associated genes (including *Wnt4*, *Tnnc2*, *Tnnt3*, and *Srl*) and slow/type1/oxidative fiber associated genes (including *Sox6*, *Tnnc1*, *Tnni1*, and *Tnnt1*). By qRT-PCR analysis, the data revealed strongly decreased gene expression of slow muscle genes after interference *MYH1B* (Fig. 6M). However, knockdown *MYH1B* had no significant expression difference in fast muscle genes (Fig. 6N). Combined, we argue that *MYH1B* not only plays a vital role in myoblast development but also in the transformation of muscle fibers.

### LncRNA-FKBP1C influence muscle fiber conversion and hypertrophy in vivo

**Fig. 7:**
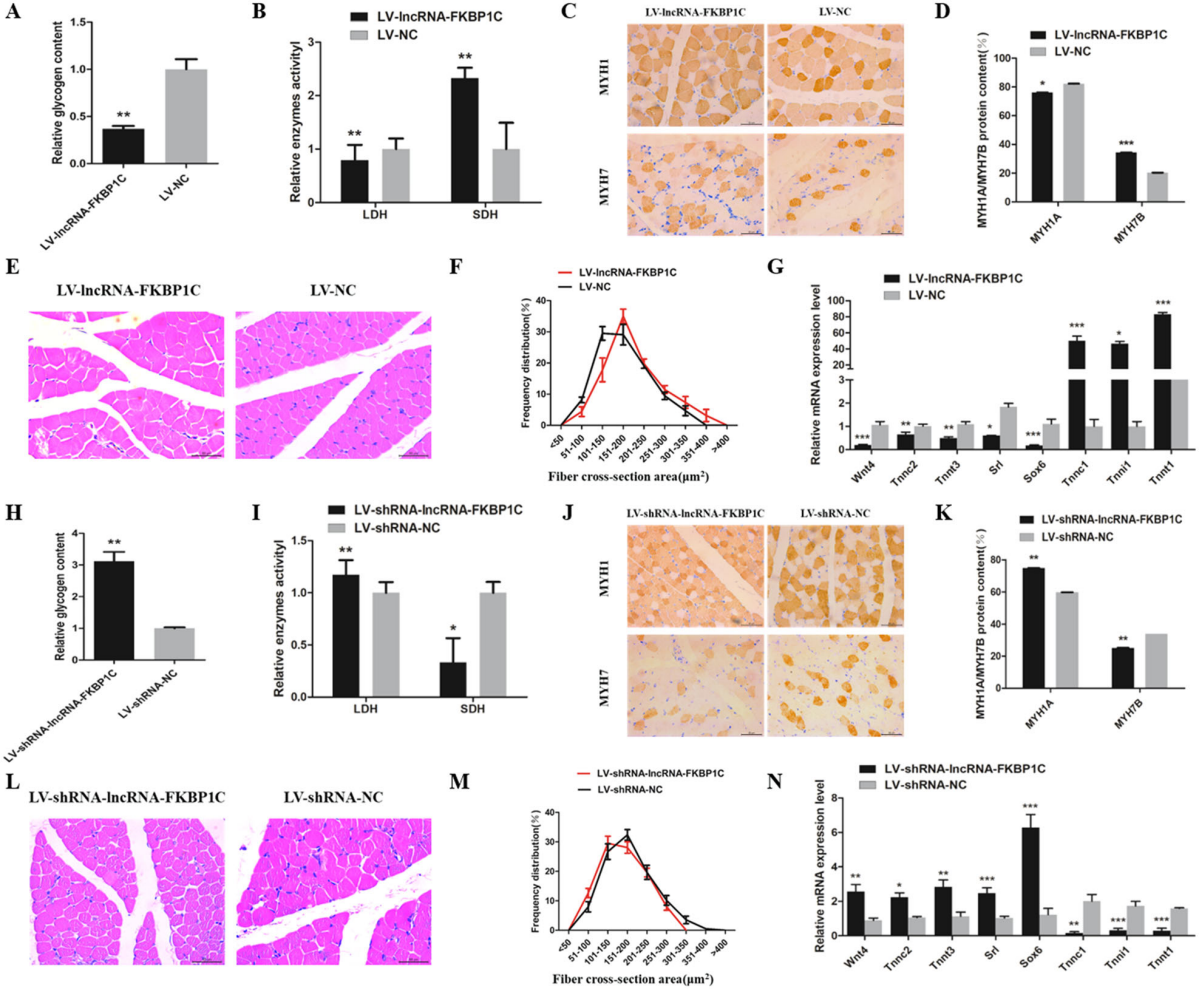
LncRNA-FKBP1C regulates muscle hypertrophy and conversion of muscle fiber types. **A** The glycogen content after lncRNA-FKBP1C overexpression prepared with lentivirus and intramuscularly injected into the gastrocnemius muscle. **B** The enzyme activity of LDH and SDH. **C** Immunohistochemistry analyses of gastrocnemius muscle fiber infected with LV-lncRNA-FKBP1C or LV-NC. **D** The data are showed as MYH1A and MYH7B protein content. **E** Hematoxylin and eosin (H&E) staining of gastrocnemius muscle. **F** The distribution of muscle fiber cross sectional area (*n* = 6 per group). **G** The mRNA level of several fast muscle genes and slow muscle genes. **H** The glycogen content after infected with LV-shRNA-lncRNA-FKBP1C or LV-shRNA-NC. **I** The enzyme activity of LDH and SDH. **J** Immunohistochemistry analyses of gastrocnemius muscle fiber. **K** The MYH1A and MYH7B protein content. **L** Hematoxylin and eosin (H&E) staining of gastrocnemius muscle. **M** The distribution of muscle fiber cross sectional area (*n* = 6 per group). **N** The mRNA level of several fast muscle genes and slow muscle genes. Results are shown as mean ± S.E.M. and the data are representative of at least three independent assays. Independent sample t-test was used to analyze the statistical differences between groups (*P < 0.05, **P < 0.01, ***P < 0.001).

To further prove that lncRNA-FKBP1C could affect the conversion of skeletal muscle fiber types in vivo, we performed lentiviral infection on the gastrocnemius muscles of 1-day-old chicks (including overexpression and interference of lncRNA-FKBP1C). Glycogen content was significantly decreased with overexpression of lncRNA-FKBP1C, and the opposite result was shown in the interference lncRNA-FKBP1C group (Fig. 7A, H). Overexpression of lncRNA-FKBP1C in vivo inhibited the enzyme activities of lactate dehydrogenase (LDH) and significantly increased the enzyme activities of succinate dehydrogenase (SDH), while knockdown of lncRNA-FKBP1C enhanced enzyme activities of LDH and decreased enzyme activities of SDH (Fig. 7B, I). The results of immunohistochemical staining of MYH1A and MYH7B showed that lncRNA-FKBP1C could drove the transformation of fast-twitch muscle fibers to slow-twitch muscle fibers (Fig. 7C, D, J, and K). Besides, we detected the expression of fast-type and slow-type muscle fiber associated genes in vivo. We found that the results were consistent with those in the CPM cells (Fig. 7G, N). Moreover, the frequency distribution of muscle fiber cross-sectional area with overexpression and interference of lncRNA-FKBP1C showed that lncRNA-FKBP1C could promote muscle fiber hypertrophy (Fig. 7E, F, L, and M). We conclude that lncRNA-FKBP1C is associated with muscle fiber conversion and hypertrophy in vivo.

## Discussion

It is well known that lncRNAs can act as an important regulatory factor in myogenesis^22–24,33–38^. For instance, lncRNA-SYISL was reported to function as a repressor of muscle development and play a vital role in muscle development mediated by PRC2^24^. In this present study, we first reveal the function of lncRNA-FKBP1C in myoblast proliferation and differentiation. Also, lncRNA-FKBP1C is involved in the formation of skeletal muscle fibers. Overexpression of lncRNA-FKBP1C represses cell proliferation and promotes cell differentiation in myoblasts while showing a down-regulation of fast muscle genes expression and up-regulation of slow muscle genes expression. To further explore the role of lncRNA-FKBP1C in skeletal muscle fiber type conversion, we injected lentivirus into the living gastrocnemius muscle. All data showed that overexpression of lncRNA-FKBP1C could inhibit fast muscle fibers type formation and promote slow muscle fibers type formation. In summary, lncRNA-FKBP1C divers the transformation of fast-twitch muscle fibers to slow-twitch muscle fibers. Some reports suggest that the composition of muscle fiber types may influence meat quality by affecting the content of metabolites postmortem in livestock^11–13,39,40^, such as pH, meat color, and drip loss^41^. Therefore, our findings can provide new ideas for improving poultry meat quality.

Some lncRNAs were reported to regulate neighboring gene expression in *cis*, while more and more lncRNAs were found leaving the transcription site and functioning in *trans*. Trans-acting lncRNAs may interact with and regulate the expression of proteins as a molecular decoy^28,42,43^. Therefore, we presume that lncRNA-FKBP1C functions in *trans*. The lncRNA-FKBP1C was found to associate with MYH1B to enhance its protein stability. However, the underlying mechanisms of how lncRNA-FKBP1C regulates the ubiquitination or ubiquitination associated enzymes require further investigation.

*MYH3* is indispensable in the development of skeletal muscle and heart. A lot of reports suggest that mutations of *MYH3* can contribute to human bone or muscle-related diseases, such as Atrial septal defect (ASD), Sheldon-Hall syndrome (SHS), and Spondylocarpotarsal synostosis syndrome (SCTS)^44–46^. In addition, recent research reported that *MYH3* could promote slow muscle fibers but have no significant effect on fast muscle fibers in mice^33^. In our current study, we found that *MYH1B* could induce the slow-twitch muscle phenotype in chicken, as same as the function of *MYH3* in mice. Also, *MYH1B* represses cell proliferation and promotes cell differentiation in myoblasts.

Collectively, the results of our study indicate that lncRNA-FKBP1C can interact directly with MYH1B to enhance its protein stability, thus influencing myoblast proliferation and differentiation, as well as regulating the transformation of skeletal muscle fiber types.

## Materials and methods

### Ethics statement

All animal experiments in this study were conducted in strict accordance with the regulations for the Administration of Laboratory Animals of Guangdong Province. These experiments (approval number: SCAU#2020C030) were carried out under the approval of the Institutional Animal Care and Use Committee at the South China Agricultural University (Guangzhou, China). We made every effort to reduce the suffering of animals.

### Experimental animals and tissues

Four 7-week-old Xinghua female chickens were received from the Zhicheng Poultry Breeding Co., Ltd. (Guangdong, China). The tissues were collected, quickly frozen into liquid nitrogen, and then stored at −80 °C, including the cerebrum, cerebellum, hypothalamus, heart, liver, spleen, lung, kidney, breast muscle, and leg muscle.

### Cell culture

The chicken primary myoblasts (CPM) were isolated from leg muscle at the gestational age of 11 (Zhuhai Yuhe Co., Ltd., China). First, the leg muscle tissues were cut into pieces after removing the skin and bone and then digested with pancreatin containing 0.25% EDTA for 15–20 min at 37 °C. Next, the digestion was terminated with Roswell Park Memorial Institute (RPMI)-1640 medium (Gibco, USA) containing 20% fetal bovine serum (FBS, Gibco), and the digested tissue fluid was filtered through a sterile filter. The single cells were collected by centrifugation at 1500 r/min for 5 min. Afterward, chicken primary myoblasts were obtained by the “differential adhesion method”. We cultured CPM cells in RPMI-1640 medium (Gibco, USA) with 20% FBS, 0.2% penicillin, 0.2% streptomycin, and then reduced FBS in the medium to 5% to induce differentiation.

DF-1 cells were from previously frozen chicken embryo fibroblasts, cultured in Dulbecco’s modified Eagle’s medium (DMEM; Gibco) with 10% FBS, 0.2% penicillin, 0.2% streptomycin. All cells were cultured at 37 °C in a 5% CO2 cell incubator.

### RNA isolation, complementary DNA(cDNA) synthesis, and real-time (RT) PCR analysis

Total RNA was extracted from tissues or cells using RNAiso plus reagent (TaKaRa, Japan). After measuring the optical density at a Nanodrop 2000c spectrophotometer (Thermo, Waltham, MA, USA), the qualified total RNA was stored at −80 °C. The cytoplasmic and nuclear cell lysates were obtained by using a PARIS Kit (Ambion, Life Technologies, USA) according to the manufacturer’s protocol. cDNA synthesis was obtained by using a PrimeScript RT Reagent Kit with gDNA Eraser (Perfect Real Time) (Takara, Japan). Real-time quantitative PCR (qRT-PCR) reactions were performed on a QuantStudio 5 Real-Time PCR Systems (Thermo Fisher, Waltham, MA, USA) by using an iTaq Universal SYBR Green Supermix Kit (Bio-Rad Laboratories Inc., USA). qRT-PCR data were analyzed by the comparative 2^−ΔΔCT^ method^47^. The primers used for qRT-PCR in this study were listed in Supplementary Table S1.

### Rapid-amplification of cDNA ends (RACE)

The partial lncRNA-FKBP1C sequence was obtained from our previous lncRNA-seq data (accession number GSE58755). RACE PCR was carried out to obtain the full-length sequence of the lncRNA-FKBP1C. Total RNA from breast muscle tissue was used as the template for nested-PCR reactions using a SMARTer RACE cDNA Amplification Kit (Clontech, Osaka, Japan), according to the manufacturer’s instructions. The products of the RACE PCR were cloned into the pMD18-T cloning vector (PCR Cloning Kit; Fermentas, Glen Burnie, MD, USA) and sequenced by Tsingke Biotech (Guangzhou, China). Primers used for RACE PCR were listed in Supplementary Table S2.

### Plasmid construction and transfection

Eleven ORFs of lncRNA-FKBP1C were amplified and cloned into pcDNA3.1-3xFlag (SiDanSai, Shanghai, China), and pcDNA3.1-3xFlag-β-actin was used as a positive control. All primers used in this study were designed using Premier Primer 5.0 software (Premier Biosoft International, Palo Alto, CA, USA) or OLIGO Primer Analysis Software Version 7 (Molecular Biology Insights, USA), and synthesized by Tsingke Biotech (Guangzhou, China). Primers used for vector construction were listed in Supplementary Table S2.

Lipofectamine 3000 reagent (Invitrogen, USA) was used for transfections in this study, according to the manufacturer’s protocol. To interfere with lncRNA-FKBP1C, si-lncRNA-FKBP1C and ASO-lncRNA-FKBP1C were co-transfected into CPM, named as si-ASO-lncRNA-FKBP1C in this study. The transfection concentrations of siRNAs and ASO were 100 nM. All the siRNAs and ASO were obtained from RiboBio (Guangzhou, China) and the sequences were shown in Supplementary Table S3.

### EdU (5-ethynyl-2′-deoxyuridine) assay

At 48 h after transfection, the CPM cells were incubated for 2 h in 50 μM 5-ethynyl-2′-deoxyuridine (EdU; RiboBio, China). And then the cells were fixed in 4% paraformaldehyde for 30 min and stained with a C10310 EdU Apollo In Vitro Imaging Kit (RiboBio, China). The ratio of the number of EdU-stained cells to the number of Hoechst 33342-stained cells was determined using images of three randomly selected fields obtained with a fluorescence microscope (TE2000-U; Nikon, Japan).

### Flow cytometric analysis

At 48 h after transfection, the CPM cells were collected and fixed in 70% ethanol overnight at −20 °C. The flow cytometry analysis was performed on a BD AccuriC6 flow cytometer (BD Biosciences, USA) by using a Cell Cycle Analysis Kit (Thermo Fisher Scientific, Waltham, MA, USA) and data were processed using FlowJo7.6 software (Treestar Incorporated, Ashland, OR, USA).

### CCK-8 assays

The CPM cells cultured in a 96-well plate were transfected, and the proliferation of the cell culture was monitored at 12 h, 24 h, 36 h, and 48 h using the TransDetect CCK (TransGen Biotech, Beijing, China). The cells were added 10 μL of CCK solution and incubated in 1 h, using a Model 680 Microplate Reader (Bio-Rad) to measure the absorbance at 450 nm.

### Western blotting assay

The CPM cellular proteins and muscle tissues were extracted using radio-immunoprecipitation assay (RIPA) buffer and phenylmenthanesulfonyl fluoride (PMSF) protease inhibitor. The Western blot assays were carried out as previously reported^48^. The antibodies used for Western blots were as follows: Flag tag polyclonal antibody (20543-1-AP; Proteintech, USA; 1:1000), MyHC mouse monoclonal antibody (B103; DHSB, Lowa City, IA, USA; 1:500), rabbit anti-heavy chain Myosin (ab124205; Abcam, Cambridge, UK; 1:1000), HRP-conjugated monoclonal mouse anti-glyceraldehyde-3-phosphate dehydrogenase (GAPDH) (KC-5G5; Kangchen, China; 1:10,000). HRP conjugated goat anti-rabbit IgG (A21020; Abbkine, USA; 1:10,000) and HRP conjugated goat anti-mouse IgG (A21010; Abbkine, USA; 1:10,000) were used as secondary antibodies.

### Immunofluorescence

The CPM cells cultured in 12-well plates were treated with 4% formaldehyde for 20 min after 48 h of transfection. These experiments were carried out as previously reported^49^. And images of immunofluorescence were captured with a Leica DMi8 fluorescent microscope (Leica, Wetzlar, Germany). The percentage of the total image area covered by myotubes as the total myotube area was calculated by using the Image J software (National Institutes of Health, Bethesda, MD, USA).

### RNA pull-down assay, mass spectrometry, and RNA immunoprecipitation

The experiments were performed as previously reported^50^.

### Lentiviral vector infection

Three intramuscular injections of 1-day-old chicks were injected lentivirus into the gastrocnemius muscle at a dosage of 1 × 10^6^ IU/ mL (single dose injection started at Days 1, 5, and 9).

### Histology image analysis and immunohistochemical analysis

Gastrocnemius muscle tissues of 14-day-old chicken were collected from LV-lncRNA-FKBP1C, LV-NC, LV-shRNA-lncRNA-FKBP1C, and LV-shRNA-NC infected muscle (*n* = 10/case). After fixed with 4% paraformaldehyde, tissues were paraffin-embedded. Tissues sections were stained with Hematoxylin and Eosin(H&E) and then subjected to image analysis. Also, Tissue sections were subjected to immunohistochemical analysis.

Primary antibodies used for immunohistochemistry were: Mouse anti-Myosin skeletal fast (MY-32, GeneTex, 1:400 dilutions on paraffin sections); Mouse anti-Myosin heavy chain, slow contracting muscle (S58, DSHB, 1:50 dilution on paraffin sections).

### Measurement of glycogen, lactate dehydrogenase (LDH) and succinate dehydrogenase (SDH)

The chickens injected with lentivirus were fasted for 12 h before being sacrificed, and dissected gastrocnemius muscle. Glycogen, LDH and SDH were assayed using a Glycogen Content Assay Kit (Solarbio, BC0345), a Lacate Dehydrogenase (LDH) Activity Assay Kit (Solarbio, BC0685) and a Succinate Dehydrogenase (SDH) Activity Assay Kit (Solarbio, BC0955), according to the manufacturer’s instructions.

### Statistical analysis

All experimental results are presented as the mean ± S.E.M, based on at least three independent experiments. The statistical significance of differences between means was assessed by performing an unpaired Student’s *t*-test. We considered *P* < 0.05 to be statistically significant. *P < 0.05; **P < 0.01; ***P < 0.001.

## Supplementary information

Table S1

Table S2

Table S3
