# Supplementary material for: LncRNA-FKBP1C regulates muscle fiber type switching by affecting the stability of MYH1B
Source: Cell Death Discov. 2021 Apr 9;7:73. doi: 10.1038/s41420-021-00463-7 (PMC8035166; doi:10.1038/s41420-021-00463-7)
Supplement: Supplementary file 1 — Table S1 [file 41420_2021_463_MOESM1_ESM.docx]

Table S1 Primers used for real-time PCR

| Primer name | Primer sequences (5′ to 3′) | Size (bp) | Annealing temperature (◦C) |
| --- | --- | --- | --- |
| lncRNA-FKBP1C | F: AGGAAACTAAATCAAGGGTAAAGTCC  R: CAGGGTGAACAAGAACAAATCC | 178 | 61 |
| *MYH1B* | F: GCAAAGAAACAGGGAAGGTC  R: AGGCCCGAGTAGGTGTAGAT | 169 | 58 |
| *MYOG* | F: CGGAGGCTGAAGAAGGTGAA  R: CGGTCCTCTGCCTGGTCAT | 320 | 53 |
| *MYOD* | F: GCTACTACACGGAATCACCAAAT  R: CTGGGCTCCACTGTCACTCA | 200 | 53 |
| *MyHC* | F: CTCCTCACGCTTTGGTAA  R: TGATAGTCGTATGGGTTGGT | 213 | 53 |
| *Wnt4* | F: GGAGGCAGCGTTCGTCTA  R: GGCAATGTTATCGGAGCAG | 160 | 56 |
| *Tnnc2* | F: GAGCAGCAAAGATGGCGTCA  R: ATCACCGTGCCCAACTCCTT | 151 | 57 |
| *Tnnt3* | F: AGAGGGAAGAAGCAAACAGC  R: GTCCCACAGTTCCTTAGCCT | 123 | 57 |
| *Srl* | F: CCTCCTCGGGCTGGATGACA  R: GTTCTTGCTGCTTGCGGTTT | 272 | 56 |
| *Sox6* | F: TCAGGTTCAGGGTCACATGCC  R: TTGCTGGAGCTGTAAAGGGC | 208 | 56 |
| *Tnnc1* | F: GTTGAGCAGTTGACAGAAGA  R: GAACCATCATAACAAGGAAC | 223 | 57 |
| *Tnni1* | F: GAGGAGTGGGAGCAGGAGAT  R: TTCGTCCACAATCTCAACCT | 231 | 57 |
| *Tnnt1* | F: GAGCCGCACGGAGAAGGAGC  R: CCCGAAGTGGGGCATGTTGG | 139 | 57 |
| *GAPDH* | F: TCCTCCACCTTTGATGCG  R: GTGCCTGGCTCACTCCTT | 146 | 50-60 |
