# Supplementary material for: LncRNA-FKBP1C regulates muscle fiber type switching by affecting the stability of MYH1B
Source: Cell Death Discov. 2021 Apr 9;7:73. doi: 10.1038/s41420-021-00463-7 (PMC8035166; doi:10.1038/s41420-021-00463-7)
Supplement: Supplementary file 2 — Table S2 [file 41420_2021_463_MOESM2_ESM.docx]

Table S2 Primers used for RACE PCR and vector construction.

| Primer name | Primer sequences (5′ to 3′) | Size (bp) | Annealing temperature (◦C) |
| --- | --- | --- | --- |
| lncRNA-FKBP1C-5′RACE | CCTTTGGCAGGAGAAGCACA |  | Step down |
| lncRNA-FKBPIC-3′RACE-outer | GGGCTGGAGCAGGTCTGACGTGACGGA |  | Step down |
| lncRNA-FKBP1C-3′RACE-inner | AGCAGCCTGCGGTTACAAGA |  | 60 |
| lncRNA-FKBP1C-ORF-1 | F: GGG**GTACCCCATG**GGCACACAGCAGCTC  R: CGG**AATTCCG**GTCCAGCACCTGCTC | 156 | 56 |
| lncRNA-FKBP1C-ORF-2 | F: GGG**GTACCCCATG**TCATGCCAGCCCTG  R: CGG**AATTCCG**AGAGCCTTTTCCATCAGCC | 378 | 56 |
| lncRNA-FKBP1C-ORF-3 | F: GGG**GTACCCCATG**CCAGCCCTGCAAGGGGT  R: CGG**AATTCCG**CGTGTCTCTGTCGCCACTG | 180 | 56 |
| lncRNA-FKBP1C-ORF-4 | F: GGG**GTACCCCATG**GATGGGCAGACAGCCCT  R: CGG**AATTCCG**AGAGCCTTTTCCATCAGCC | 339 | 56 |
| lncRNA-FKBP1C-ORF-5 | F: GGG**GTACCCCATG**GGCAGACAGCCCTGGTG  R: CGG**AATTCCG**GCTCCCGCCGGGAGCA | 240 | 56 |
| lncRNA-FKBP1C-ORF-6 | F: GGG**GTACCCCATG**CTGCGTGCGTCAGGCTCC  R:CGG**AATTCCG**GGGGAGCTGGCACTGA | 510 | 56 |
| lncRNA-FKBP1C-ORF-7 | F: GGG**GTACCCCATG**GAGCAGGGATTTGTTCTT  R:CGG**AATTCCG**GGGGAGCTGGCACTGA | 471 | 56 |
| lncRNA-FKBP1C-ORF-8 | F: GGG**GTACCCCATG**CTGCCACACTCGTATGACA  R: CGG**AATTCCG**GAAATACAGACACCAG | 570 | 56 |
| lncRNA-FKBP1C-ORF-9 | F: GGG**GTACCCCATG**CTGTGCGTGCAGTCCT  R: CGG**AATTCCG**GAAATACAGACACCAG | 450 | 56 |
| lncRNA-FKBP1C-ORF-10 | F: GGG**GTACCCCATG**CCCTGCACAGAGCTCA  R: CGG**AATTCCG**CCGATCCCCAAGCGAGG | 162 | 56 |
| lncRNA-FKBP1C-ORF-11 | F: GGG**GTACCCCATG**TGCCCTGGGCTGGCG  R: CGG**AATTCCG**CCGATCCCCAAGCGAGG | 132 | 56 |
| pcDNA3.1-3xFlag-β-actin | F: GGG**GTACCCCATG**ATGGATGATGATATTGCTGC  R: CGG**AATTCCG**AAAAGACACTTGTTGGGTTAC | 1548 | 58 |
