# Supplementary material for: LncRNA-FKBP1C regulates muscle fiber type switching by affecting the stability of MYH1B
Source: Cell Death Discov. 2021 Apr 9;7:73. doi: 10.1038/s41420-021-00463-7 (PMC8035166; doi:10.1038/s41420-021-00463-7)
Supplement: Supplementary file 3 — Table S3 [file 41420_2021_463_MOESM3_ESM.docx]

Table S3 siRNAs and ASO used for RNA interference.

| Fragment name | Sequence (5′ to 3′) |
| --- | --- |
| si-gga-lncRNA-FKBP1C | CTAGCATTGCTCTCTAGAT |
| ASO-gga-lncRNA-FKBP1C | CTTCCCACAGCTCCTGCACA |
| si-gga-MYH1B | GTTTCATGGCAAGAAGATA |
